# Phenotypic changes in the brain of SIV-infected macaques exposed to methamphetamine parallel macrophage activation patterns induced by the common gamma-chain cytokine system

**DOI:** 10.3389/fmicb.2015.00900

**Published:** 2015-09-14

**Authors:** Nikki Bortell, Brenda Morsey, Liana Basova, Howard S. Fox, Maria Cecilia Garibaldi Marcondes

**Affiliations:** ^1^Department of Molecular and Cellular Neurosciences, The Scripps Research InstituteLa Jolla, CA, USA; ^2^Department of Pharmacology and Experimental Neuroscience, University of Nebraska Medical CenterOmaha, NE, USA

**Keywords:** gamma-chain cytokines, SIV, HIV infections, NeuroAIDS, methamphetamine, IL15, macrophages, microglia

## Abstract

One factor in the development of neuroAIDS is the increase in the migration of pro-inflammatory CD8 T cells across the blood–brain barrier. Typically these cells are involved with keeping the viral load down. However, the persistence of above average numbers of CD8 T cells in the brain, not necessarily specific to viral peptides, is facilitated by the upregulation of IL15 from astrocytes, in the absence of IL2, in the brain environment. Both IL15 and IL2 are common gamma chain (γc) cytokines. Here, using the non-human primate model of neuroAIDS, we have demonstrated that exposure to methamphetamine, a powerful illicit drug that has been associated with HIV exposure and neuroAIDS severity, can cause an increase in molecules of the γc system. Among these molecules, IL15, which is upregulated in astrocytes by methamphetamine, and that induces the proliferation of T cells, may also be involved in driving an inflammatory phenotype in innate immune cells of the brain. Therefore, methamphetamine and IL15 may be critical in the development and aggravation of central nervous system immune-mediated inflammatory pathology in HIV-infected drug abusers.

## Introduction

The immunological environment of the central nervous system (CNS) is highly susceptible to changes due to a number of insults, such as infections that cross the blood–brain barrier (BBB), or even related peripheral changes. In the case of infections with HIV in humans, or simian immunodeficiency virus (SIV) in macaques, the viruses are able to reach the brain very early. As a consequence, glial activation and the development of a pro-inflammatory cytokine environment favor the accumulation of activated CD8 T cells and macrophages (Marcondes et al., 2001, 2007). These changes in the brain are associated to the development of deficits in motor and cognitive abilities, and other disruptions such as in the circadian rhythm of body temperature and sleep patterns (Marcondes et al., 2001; Huitron-Resendiz et al., 2007).

Drugs of abuse can also alter the levels of inflammatory factors in the brain. Methamphetamine (Meth), for instance, which is an illegal psycho-stimulant abused at epidemic proportions in USA and worldwide (Mathers et al., 2008) can exacerbate inflammation in the CNS (Mathers et al., 2008). Furthermore, a large number of HIV-infected individuals acquire the virus from Meth use or are exposed to this drug during disease course (Mathers et al., 2008). Both HIV infection and Meth dependence can be associated with brain dysfunction. In fact, viral burden in the brain and inflammatory neuropathology associated with HIV infection are drastically aggravated by Meth use (Everall et al., 2005), which contributes to the severity of neuroAIDS.

In an experimental model of Meth-abuse in simian immunodeficiency virus (SIV)-infected macaques, we have previously found that Meth treatment aggravated the inflammatory pathology and increased viral load in the brain (Marcondes et al., 2010). We have also shown that one of the factors involved in the overall development of neuroAIDS is an increase, of up to 10-fold, in the migration of pro-inflammatory CD8 T cells (Marcondes et al., 2001). Importantly, specific CD8 T cells in the brain tissue keep viral load down (Marcondes et al., 2015). However, the persistence of CD8 T cells that are not necessarily specific to viral peptides is facilitated by the upregulation of IL15 in astrocytes, and an absence of IL2, both of which are common gamma chain (γc) cytokines (Marcondes et al., 2007). How the expression of IL15 and other cytokines of the common γc family are affected by Meth, and whether they can contribute to the enhanced pathology caused by the drug in the brain, have never been addressed.

The common gamma chain (γc; CD132), also known as interleukin-2 receptor gamma subunit or IL-2RG, is a cytokine receptor sub-unit common to receptor complexes for different interleukin receptors, including IL-2, IL-4, IL-7, IL-9, and IL-15. Each one of these cytokines use additional subunits that provide signaling and function specificity (Fehniger and Caligiuri, 2001). These cytokines play a role in both the innate and adaptive immune responses. The fact that these cytokines share γc binding provides a basis for some of their redundant actions. However, γc-cytokines may also compete for opposing effects based on their binding to other potential receptors. For instance, IL-7 regulates survival of immature and mature T lymphocytes, while IL-2, IL-15, and IL-21 appear to have specific functions in T cell homeostasis and memory differentiation; on the other hand, the balance between IL2 and IL15 deeply affects T cell survival (Marrack et al., 1998; Vella et al., 1998; Boyman et al., 2007; Mitchell et al., 2010). Memory CD8 T cells can utilize either IL-7 or IL-15 to undergo Ag-independent homeostatic proliferation and activation (Tan et al., 2002). Conversely, HIV gag-specific T cells show enhanced Ag-specific IFN-γ responses upon exposure to IL-2, IL-15, or combined IL-15/IL-7, suggesting that these cytokines are able to rescue activation in specific cells (Gu et al., 2007), as well as in cells with other specificities.

The effects of γc-cytokines in cells of the innate immune system are not well-known. Interestingly, γc-cytokines, especially IL15, affect the development and function of NK cells (Kawamura et al., 2003). It is also known that IL15 induces proliferation of innate immune cells, and signals via IL-15Rα, JNK, and NF-κB to drive RANTES production by myeloid cells (Chenoweth et al., 2012). In human monocytes, IL-15 induces IL-8 and MCP-1 production, which further attract inflammatory cells to expression sites (Badolato et al., 1997). In addition, the interaction of IL-15-IL-15R is critical in early activation of antigen-presenting cells and plays an important role in the innate immune system (Ohteki et al., 2001). In macrophages, IL-15 also functions as a potent autocrine regulator of proinflammatory cytokine production by these cells, with high concentrations of IL-15 favoring TNF-α, IL-1, and IL-6 production, and very low concentrations of IL-15 favoring IL-10 production (Alleva et al., 1997).

Here, we investigated the expression of γc-cytokines and receptors in isolated mononuclear cells from the brains of SIV-infected macaques, treated or not with Meth, in an effort to explain the development of a pro-inflammatory environment and high NK activation. We also examined the ability of IL15 to induce an innate immune phenotype that mimics the one found in Meth use. Our results suggest a role for IL15 and the common gamma chain system in the inflammatory pathogenesis of SIV, which is aggravated by Meth in the CNS.

## Materials and Methods

### Monkeys and SIV Infection

SIV, SRV-type D, and Herpes B Virus-free rhesus macaques of Chinese origin purchased from Valley Biosystems (West Sacramento, CA, USA), were infected with a cell-free SIV stock derived from SIVmac251 (Lane et al., 1995; Watry et al., 1995). All animal experiments were performed with approval from the Institutional Animal Care and Use Committee and followed NIH guidelines. Animals kept in containment were anesthetized with 10–15 mg/kg of ketamine intramuscularly prior to experimental procedures. Blood was serially drawn from the femoral vein, and plasma was obtained from EDTA-treated blood. At necropsy, which was performed after terminal anesthesia, animals were intracardially perfused with sterile PBS containing 1 U/ml heparin. Tissue samples were taken for cell isolation, virus quantification, and formalin fixation for histology.

### Meth Treatment and Design

At 19 weeks pi, when plasma viral load averaged 5.3 (log10), three out of seven infected animals were started on an escalating protocol of Meth administration. Importantly, we mimicked the amount of Meth used by chronic abusers with our drug administration schedule. Matching uninfected controls were also included. Human chronic Meth abusers, who generally increase their dosage over time, reach up to 1 g/day. In a recent high-resolution MRI study demonstrating Meth-induced defects in human brains, subjects averaged 3.4 g/week of Meth (Thompson et al., 2004). Assuming an average of 70 kg body weight and 50% purity of the Meth (the average according to US Department of Justice statistics), this averages to ∼24–25 mg/kg/week. Since a typical abuser takes Meth 1–3 times a day for more than 20 days a month (Simon et al., 2002), we used a modification of our escalating dose regimen, giving Meth for 5 days a week, twice a day, at a dose of 2.5 mg/kg. After a 5 weeks ramp-up, final total dosage was 25 mg/kg/week, approximating the amounts used by chronic Meth abusers. This level was then maintained for an additional 18 weeks. The four uninfected control animals, and four SIV-infected animals, received PBS injections on the same schedule. All animals were sacrificed at 42 weeks pi.

### Mononuclear Cells

The macaque brain was removed at necropsy following intravascular perfusion. For isolation of cells from the brain, the brain was carefully freed of meninges. Brain mononuclear cells were isolated by enzymatic digestion of minced tissue, followed by Percoll (Sigma-Aldrich) gradient, as previously described (Marcondes et al., 2001). Cell pellets were either stained with antibodies for flow cytometry or snap frozen and maintained in liquid nitrogen prior to mRNA extraction either for gene array or for real time PCR.

### Flow Cytometry

Percoll-isolated mononuclear cells from macaque brains as well as THP1 macrophages from cell culture (see below) were washed in PBS containing 2% FCS and 0.01% NaN3, and characterized by staining with labeled antibodies, using BD Cytofix/Cytoperm solution kit (BD Pharmingen, San Jose, CA, USA), For cell surface phenotyping and proliferation, the antibodies used were: PE-labeled anti-CD11b clone M1/70 (Biolegend, San Diego, CA, USA), APC-labeled anti-human CD195 (CCR5), clone J418F1 (Biolegend), PeCy7-labeled anti-human CD11a clone 25.3.1 (Beckman Coulter, Brea, CA, USA), and FITC-labeled anti-Ki67 clone MIB-1 (Dako, Carpinteria, CA, USA). Stained cells were acquired by a FACSCalibur (BD Biosciences, San Jose, CA, USA) flow cytometer, and analyzed in FlowJo 6.2.1 software (Tree Star, Inc., San Carlos, CA, USA).

### Gene Arrays and Pathway Analysis

Gene analysis was performed by Miltenyi Biotec. All samples were individually performed in duplicate. RNA was isolated from 17 macaques using standard RNA extraction protocols (NucleoSpin RNA II, Macherey-Nagel). Quality of the samples was checked via Agilent 2100 Bioanalyzer platform (Agilent technologies). A RNA integrity number (RIN) was calculated by a proprietary algorithm that takes several QC parameters into account, such as 28S/18S RNA peak area ratios and unexpected peaks in the 5S RNA region, and RIN number of 10 indicates high quality, and 1 low quality. A RIN >6 is of sufficient quality for gene expression profiling experiments (Fleige and Pfaﬄ, 2006; Fleige et al., 2006). All samples, except for one animal (492 – Normal control) showed values above 6. That animal was excluded from the analysis, leaving the healthy control group with an *n* = 4. For the linear T7-based amplification step, 100 ng of each total RNA sample was used. To produce Cy3-labeled cRNA, the RNA samples were amplified and labeled using the Agilent Low Input Quick Amp Labeling kit (Agilent Technologies) following the manufacturer’s protocol. Yields of cRNA and the dye incorporation rate were measured with the ND-1000 spectrophotometer (Nanodrop Technologies). The hybridization procedure was performed according to Agilent 60-mer Oligo microarray processing protocol using the Agilent gene expression hybridization kit (Agilent Technologies). Briefly, 1.65 ug Cy3-labeled fragmented cRNA in hybridization buffer was hybridized overnight (17 h, 65°C) to Agilent Whole Rhesus monkey Genome oligo microarrays 4x44K (one-color) using Agilent’s recommended hybridization chamber and oven. Finally, the microarrays were washed once with the Agilent Gene expression wash buffer 1 for 1 min at room temperature, followed by a second wash with pre-heated Agilent Gene expression wash buffer 2 (37°C) for 1 min. The last washing step was performed with acetonitrile. Fluorescent signals of the hybridized microarrays were detected using Agilent’s microarray Scanner System (Agilent Technologies). The Agilent Feature extraction software (FES) was used to read out and process the microarray image files. The software determines feature intensities (including background subtraction), rejects outliers and calculates statistical confidences. For determination of differential gene expression, FES derived output data files were further analyzed using Rosetta Resolver gene expression data analysis system (Rosetta Biosoftware). This software offers the possibility to compare two single intensity profiles in a ratio experiment. Output data were normalized by dividing their intensity values by the median. These normalized signal intensities were joined to a common table with the entire normalized experiment data list. Fold change, sample/control log10 ratios, and p values were analyzed with Resolver software. The complete expression dataset was then mapped and groups were compared using an integrative systems biology approach. To complete the bioinformatics analysis, two knowledge base resources were queried: the Ingenuity Knowledge Base (Calvano et al., 2005) and an interaction repository, which is based on cpath (Cerami et al., 2006, 2011; Cline et al., 2007), and includes interactions curated by GeneGo (http://www.genego.com) and Ingenuity. We focused on brain-derived mononuclear cells to compare between Meth and Control and between SIV/Meth and SIV, to select for high score gene nodes, filtered for a minimum of three genes showing similar behavior.

### Real Time PCR

First Strand kit (Qiagen) was used for cDNA synthesis. Primers in **Table 1** were designed based on available sequences for Macaca mulatta in Gene Database for detection of relative levels using SyBrGreen/ROX in an ABI HT7900 machine. Data was analyzed with Sequence Detection System software and normalized with GAPDH expression.

**Table 1:** Macaque primer sequences.

	Forward 5′	Reverse 3′
*rhIL2*	gtcacaaacagtgcacctac	atggttgctgtctcatcagc
*rhIL4*	tgcctccaagaacacaactg	aacgtactctggttggcttc
*rhIL7*	cgcaagttgaggcaatttct	ctttgttggttggggttcac
*rhIL21*	tcgccacatgattagaatgc	gcacctgtggaaggtgattt
*rhIL2RG*	gtcagtgagattcccccaaa	atggggacacaaaattccaa
*rhITK*	tctgggaattagccccttct	taggcattctcgtgcctctt
*rhIL7R*	gcgtatgtcaccatgtccag	aggatgctcttgcctcatgt
*rhIL15RA*	gatgccttccacatcgtctt	ttgatgtagcatgccaggag
*rhIL21R*	cgtggtgtccatcgatacag	gggtggctttagtctgtcca
*rhNOS2*	acacctcaccacaaggccaa	ctggggaacaagacaggcat
*rhIL6*	ccagccactgacctcttcag	gagatgcgtcgtcatctcct
*rhMrc1*	caccaaaacctgagccaacg	ggcccaagacacgtaatcca
*rhCD163*	gcaggttctggacgcatttg	acacactgttccccactgtc
*rhArg1*	ttggcttgagagacgtggac	gagcaactccaaagcaagcc
*rhStat6*	ttgaactcgctggacagagc	aagtcgacatagagccgctg

### Immunohistochemistry

Macaque tissue was fixed in 10% formalin and paraffin embedded. Five μm sections were stained with hematoxylin and eosin and examined microscopically. Indirect immunohistochemical staining for IL15 (R&D Systems), NOS2 (Cayman Chemical, Ann Arbor, MI, USA), IL6 and CD163 (both Novus Biologicals, Litleton, CO, USA), were performed as previously described (Roberts et al., 2003; Marcondes et al., 2007). Colorimetric development was performed with the NovaRed chromagen (Vector) followed by a hematoxylin counterstain (Sigma-Aldrich).

### THP1 Cell Culture and Stimulation

THP1 cells were maintained in our lab, in suspension, in cell culture flasks (Costar, Cambridge, MA, USA) at a density of 2.5 × 10^5^ cells/ml to 1.0 × 10^6^ cells/ml in RPMI-1640 with GlutaMAX containing 10% fetal calf serum (Invitrogen, Carlsbad, CA, USA), 10 mM HEPES (Invitrogen), and Pen-Strep (Invitrogen). Cells were plated at 1 × 10^6^ cell/ml in 24 well flat-bottomed plates (Costar) and treated with the cytokines IL4 (10 U/ml), IL7 (10 U/ml), IL15 (50 U/ml), or with the control IFNb (0.5 ng/ml) for 48 h (R&D Systems, Minneapolis, MN, USA). Cell viability following treatments was confirmed using Trypan Blue (Invitrogen) exclusion test. RNA extraction after treatments was completed using the RNeasy Mini Kit (Qiagen), followed by First Strand kit (Qiagen) for cDNA synthesis. Primers in **Table 2** were based on available sequences for Homo sapiens in Gene Database for detection of relative levels using SyBrGreen/ROX in an ABI HT7900 machine. Data was analyzed with Sequence Detection System software and normalized with GAPDH expression.

**Table 2:** Human primer sequences.

	Forward 5′	Reverse 3′
*HuNOS2*	gtggaagcggtaacaaagga	tgccattgttggtggagtaa
*HuTNFa*	cagagggcctgtacctcatc	ggaagacccctcccagatag
*HuIL6*	agtgcctctttgctgctttcac	tgacaaacaaattcggtacatcct
*HuArg1*	cctttctcaaagggacagcca	gatgggtccagtccgtcaaca
*HuMrc1*	ggcggtgacctcacaagtat	ttttcatggcttggttctcc
*HuCD163*	ttgccagcagttaaatgtg	aggacagtgtttgggactgg
*HuTGFb*	ctgctgaggctcaagttaaaagtg	tgaggtatcgccaggaattgtt
*HuStat6*	ctgccaaagacctgtccatt	ggtaggcatctggagctctg
*HuIL10*	gccgtggagcaggtgaag	tggctttgtagatgcctttctct

### Statistical Analysis

Group comparisons for individual genes across conditions were performed using one way ANOVA, followed by Bonferroni’s *post hoc* tests. The difference between the means was considered significant at *p* < 0.05. Tests were performed using Prism software (GraphPad Software, San Diego, CA, USA) for Macintosh.

## Results

### Expression of Molecules of the IL2RG System are Modified by Meth *In Vivo*

Immune cells isolated from macaque’s brains were highly enriched for CD11b expression (>89%), therefore mostly comprised of microglia and brain resident and infiltrating macrophages, 0.5–7% CD3+CD8+ T lymphocytes and <2% CD3–CD8+ NK cells, as previously described. These cells, denominated brain mononuclear cells, were characterized regarding changes in gene expression caused by SIV infection, Meth, or the interaction between SIV and Meth. Using gene array on the isolated mononuclear cells, followed by a systems biology analysis approach, we focused on genes that were upregulated both in the SIV-only and in Meth-only groups, compared to controls, and that were also upregulated in the SIV/Meth group compared to SIV-only group. We found that the expression of IL2RG, used by all common gamma chain cytokines, exhibited a highly significant upregulation pattern in SIV compared to control (*p* = 0.000045), and in SIV/Meth compared to SIV (*p* = 0.0001). In terms of fold change, IL2RG was among the 50 genes most upregulated by Meth alone, compared to controls (2.06-fold, *p* = 0.001). Further investigation of the gene cluster associated to the IL2RG gene was further analyzed, given their important role for the development of an inflammatory millieu. Of the cytokines that utilize the common gamma-chain, the expression of IL4 and IL15 was significantly enhanced by Meth (**Table 3**). On the other hand, and consistent with previous findings upon SIV infection (Marcondes et al., 2007), the expression of IL2 was not significantly increased by Meth either alone or in SIV-infected macaques (**Table 3**). The IL15RA, which binds IL15 with high affinity, was also upregulated by Meth, but not by SIV alone (**Table 3**). Together, these findings suggest that the molecules in the IL2RG cytokine system may are affected by Meth, and may play a role on the effects of the drug in the context of SIV infection in the brain.

**Table 3:** Fold change and statistical behavior of common yc family molecules and related signaling components from gene profiling of isolated microglia and brain mononuclear cells.

Gene name	Fold change Meth/Ctr	*p*-value Meth/Ctr	Fold change SIV/Ctr	*p*-value SIV/Ctr	Fold change SIVMeth/SIV	*p*-value SIVMeth/SIV
IL2	1.13	0.76	0.54	0.13	2.17	0.41
IL4	**2.58**	**0.016**	1.38	0.19	**15.49**	**0.03**
IL7	1.11	0.72	1.31	0.32	6.89	0.19
IL7R	1.72	0.12	1.64	0.23	**5.3**	**0.049**
IL9R	0.73	0.30	0.79	0.28	1.56	0.32
IL15	1.21	0.35	**2.01**	**0.026**	**4.73**	**0.04**
IL15RA	**1.84**	**0.05**	3.82	0.05	**2.43**	**0.05**
IL21	1.04	0.91	**0.5**	**0.01**	1.53	0.35
IL21R	0.98	0.91	4.16	0.19	2.76	0.18
IL2RG	**2.06**	**0.001**	**4.49**	**0.00005**	**6.71**	**0.0001**
ITK	1.00	0.99	1.33	0.53	**11.8**	**0.026**

*Values in bold are statistically significant.*

**FIGURE 1:**
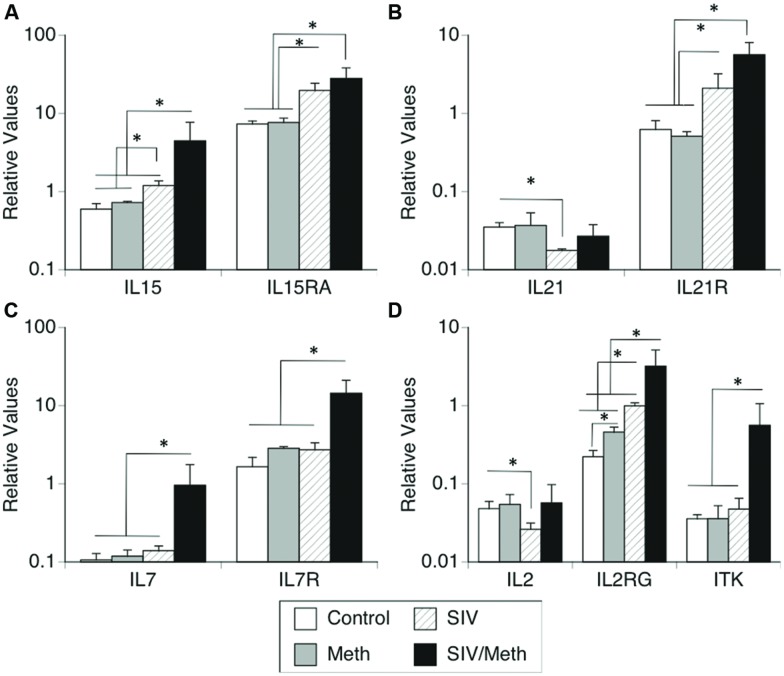
**Transcriptional levels of (A) IL15 and IL15RA, (B) IL21 and IL21R, (C) IL7 and IL7R, and (D) IL2, IL2RG and ITK.** Transcriptional expression of common gamma chain pathway components in isolated immune cells from the brain of rhesus macaques infected with SIV and treated with Meth. Results are expressed as average ± SEM of three technical replicates, with results normalized to the expression of GAPDH. ^∗^*p* ≤ 0.05 in comparisons shown in lines (one-way ANOVA *p*-value is reported in Results, and was followed by Bonferroni’s *post hoc* test).

We further confirmed changes in the expression of IL2RG, co-receptors and ligands involved in this system by using SyBr Green – qRT-PCR (**Figure 1**). Together, the expression of molecules in the IL2RG system showed equal variance (Bartlett’s *p* < 0.0001), and there was a significant difference between all groups (two-way ANOVA *p* = 0.013, *p* < 0.0001 for the effect of treatments, *p* < 0.0001 for the effect of genes, and *p* < 0.0001 for the effect of interaction). Individually, Meth only and SIV only caused differences in the transcriptional levels of these molecules. For instance, confirming our previous findings (Marcondes et al., 2007), IL15 was upregulated in the brain by SIV infection, and was further increased by Meth in SIV-infected macaques (ANOVA *p* = 0.0069; **Figure 1A**). Furthermore, the expression of IL15RA was significantly increased both by SIV infection and by SIV/Meth, compared to uninfected controls and to Meth-only controls (ANOVA *p* = 0.0001; **Figure 1A**).

The expression of IL21 was significantly decreased by SIV infection, but this expression did not differ between SIV/Meth and baseline (ANOVA *p* = 0.0001; **Figure 1B**). On the other hand, the levels of the IL21R were increased both by SIV and SIV/Meth, but no additive effect was observed (**Figure 1B**).

The expression of IL7 and IL7R in the brain was not altered either by Meth alone or by SIV alone. However, in Meth-treated SIV-infected animals, both IL7 and IL7R were significantly increased compared to controls (ANOVA *p* = 0.0122 and *p* < 0.0001, respectively; **Figure 1C**).

Confirming our previous findings in SIV-infected brains (Marcondes et al., 2007), IL2 expression was decreased by SIV infection. Interestingly, neither the Meth alone group nor the SIV/Meth group had IL2 levels that differed from baseline (ANOVA *p* = 0.049; **Figure 1D**). However, we confirmed the significant upregulation of IL2RG both by Meth and by SIV, and a significant additive effect in SIV/Meth animals (ANOVA *p* = 0.008; **Figure 1D**). Conversely, we also confirmed that the expression of ITK was increased in the brain of SIV/Meth animals, but not in Meth or SIV-only groups when compared to controls (ANOVA *p* = 0.012; **Figure 1D**).

By qRT-PCR, IL4 expression was not affected by Meth or SIV, but it was significantly upregulated in SIV/Meth animals (5.3-fold) compared to controls or to SIV alone (not shown, ANOVA *p* = 0.0157, Bonferroni *p* = 0.05 and *p* = 0.043, respectively). The expression of the IL4R was not detectable in brain mononuclear cells.

**FIGURE 2:**
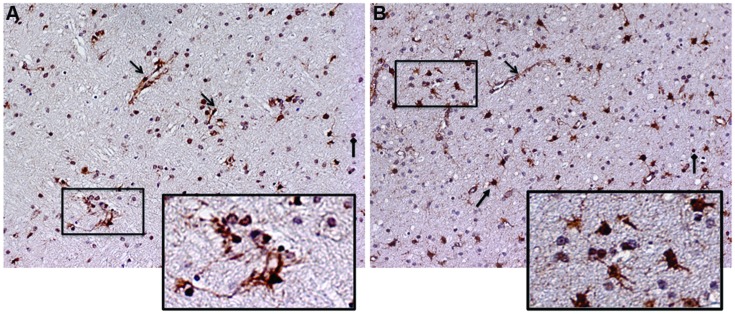
**Immunohistochemistry for detection of IL15 on frontal cortex sections from the brain of a representative (A) SIV-infected animal and of a (B) SIV-infected Meth-treated animal.** Arrows indicate different cell types exhibiting positive staining. Open-head arrows indicate endothelial cells, closed-head arrows indicate astrocytes, and square-head arrows indicate infiltrating leukocytes. Rectangles indicate magnified regions.

Together, these data validate our systems approach, and suggest that Meth can further enhance the levels of cytokines and receptors in the IL2RG pathway, both alone and in the context of SIV infection. Among these molecules, IL15 and IL15RA were significantly upregulated in the context of SIV, and were also further upregulated by Meth. Thus, we examined a potential role for IL15 in modifying inflammatory characteristics associated to pathogenesis. We investigated IL15 tissue localization using immunohistochemistry and detected an important increase at the protein level. **Figure 2** shows the staining pattern of IL15 on frontal cortex sections from SIV and SIV/Meth macaques (**Figures 2A,B**, respectively). The staining was predominantly associated with endothelial cells, astrocytes, and infiltrating cells (see arrows and legend). The increase in IL15 and in the number of IL15-expressing cells caused by Meth appeared to be mainly associated to astrocytes, but also to a higher number of infiltrating mononuclear cells with morphological characteristics of macrophages (**Figure 2B**).

### IL2RG – Binding Cytokines Increase Macrophage Proliferation

We have previously shown that the increase in IL15, which is involved in homeostatic T cell proliferation, is one of the factors responsible for the persistence of CD8 cells in the brain of SIV-infected macaques (Marcondes et al., 2007). However, although SIV-infected Meth-treated macaques presented higher levels of IL15 in the brain, they did not show a higher number of CD8 T cells in the brain compared to SIV only (Marcondes et al., 2010). Nevertheless, a higher inflammatory infiltrate, characterized by macrophages expressing activation markers such as CCR5 and high CD11b, was induced by Meth in SIV-infected brains (Marcondes et al., 2010). In addition, the isolated macaque mononuclear cells examined in this study were highly enriched for CD11b+ cells (≥89%). Therefore, we investigated whether IL15 signaling through IL2RG could potentially act on innate immune cells such as macrophages, and we also compared its performance to the performance of other IL2RG-binding cytokines that were also increased by Meth and/or SIV, IL4 and IL7. For consistency, here we used THP1 cells, which are from a stable human macrophage cell line. Human macrophages are different from microglia, but serve as a reasonable approximation for cell culture experiments and to strengthen evidence for pathways that converge with brain findings.

**FIGURE 3:**
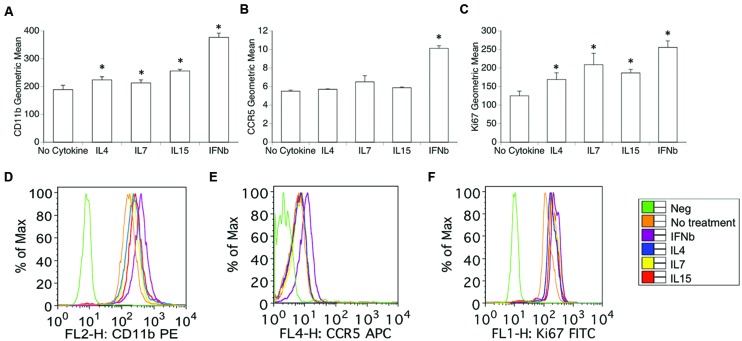
**Expression of surface activation and nuclear proliferation markers in macrophages exposed to IL2RG chain cytokines IL4, IL7, and IL15.** Geometric mean fluorescence of surface **(A)** CD11b, **(B)** CCR5, and **(C)** intracellular Ki67. Results represent the average ± SEM of three experiments performed in duplicate. Expression histograms of **(D)** CD11b, **(E)** CCR5, and **(F)** Ki-67 show the surface marker distribution in one representative experiment. Isotype controls were utilized as Negative (Green lines). THP1 cells were treated with 10 U of each cytokine or 1 ng/ml IL4, 1 ng/ml IL7, and 0.5 ng/ml IL15 for 48 h. IFNγ 0.5 ng/ml was used as a positive control. The cells were harvested and stained for surface CD11b, CCR5 and intracellular Ki67 as described. Results are expressed in average ± SD of geometric mean fluorescence. ^∗^*p* < 0.05 compared to untreated control.

Using flow cytometry, we examined whether optimal physiological concentrations of IL15 and of the other cytokines that bind to IL2RG, including IL4 and IL7, have the ability to induce changes in the surface expression of previously described activation and proliferation markers (Marcondes et al., 2001, 2007, 2008). Importantly, these treatments did not affect the viability of the cells. For this assay, IFNb was used in parallel as a positive control, and consistently induced an increase in activation, as accessed by the expression levels of CD11b, CCR5, and CD11a by flow cytometry (**Figure 3**). In forward versus side scatter plots, the cells were highly homogeneous, and were gated based on average size, to exclude potential doublets and debris. The exposure of THP1 macrophages to IL4, IL7, and IL15 over the course of 48 h increased the expression of CD11b (ANOVA, excluding IFNγ, *p* = 0.023; **Figures 3A,D**), but did not affect the expression of CCR5 (**Figures 3B,E**), or other subset markers such as CX3CR1, CCR2, and CD80 (not shown). The percentage of cells expressing the activation marker CD11a high was also increased by IL4, IL7, and IL15 (not shown, ANOVA, except IFNγ, *p* = 0.0016). Without treatment, 40.1% (±7.23) of the THP1 macrophages were CD11a high, and the percentage was increased to 53.75 (±4.15, Bonferroni *p* = 0.0001) by IL4, to 50.83 (±5.13, Bonferroni *p* = 0.03) by IL7, and to 66.83 (±3.14, Bonferroni *p* = 0.0001) by IL15. These cytokines also significantly increased the expression of Ki67 in macrophages (ANOVA, excluding IFNγ *p* = 0.001; **Figures 3C,F**), suggesting their ability to increase proliferation rates in these cells.

Together, this data suggests that the IL2RG binding cytokines can affect selected activation markers associated to activation/adhesion as well as proliferation in macrophages, without affecting subset markers.

### IL2RG Cytokines Enhance the Transcription of M1 and M2 Functional Macrophage Phenotype Markers *In Vitro*

In the THP1 cell system, we also examined whether the IL2RG cytokines can induce a functional phenotypic bias on innate immune cells. We examined the transcriptional expression of molecules that characterize M1 (NOS2, TNFα, and IL6) and M2 (Arg1, Mrc1, CD163, TGFβ, Stat6, and IL10) phenotypes.

**FIGURE 4:**
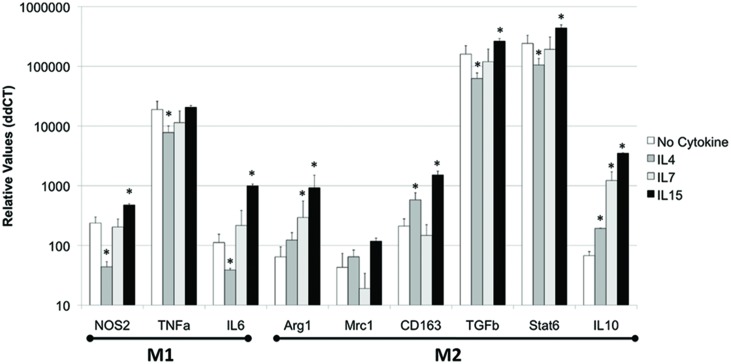
**IL4, IL7, and IL15 act differentially to modify functional macrophage phenotype *in vitro*.** THP1 cells were treated with 10 U of each cytokine or 1 ng/ml IL4, 1 ng/ml IL7, and 0.5 ng/ml IL15 for 48 h. IFNγ 0.5 ng/ml was used as a control. The cells were harvested and phenotypic markers were accessed by qPCR. Results represent the average ± SEM of three experiments performed in triplicate. ^∗^*p* < 0.05 compared to untreated control.

Regarding M1 functional markers, IL4 significantly decreased the expression of NOS2, TNFα, and IL6 (*p* = 0.031, *p* = 0.0006, *p* = 0.045, respectively), while IL7 did significantly decrease the expression of TNFα (*p* = 0.00005) compared to controls (**Figure 4**). On the other hand, IL15 significantly increased the expression of NOS2 and IL6 (*p* = 0.0005 and 0.05, respectively), but did not affect the expression of TNFα in macrophages *in vitro*, compared to no cytokine treatment (**Figure 4**).

Regarding M2 functional markers, IL4 increased the expression of CD163 and IL10, but decreased the expression of TGFβ and Stat6 (**Figure 4**). IL7 was able to induce the upregulation of only IL10 (*p* = 0.0004, **Figure 4**). On the other hand, IL15 induced the transcriptional increase of Arg1, CD163, TGFβ, Stat6, and IL10 (*p* = 0.02, *p* = 0.0003, *p* = 0.000003, *p* = 0.00002, *p* = 0.00009, respectively), but not of Mrc1, compared to controls.

Overall, IL2RG cytokines were able to affect both M1 and M2 markers in macrophages. Furthermore, while IL4 largely decreased M1 phenotype, and IL7 had a limited ability to enhance M2 markers, IL15 was able to upregulate both M1 and M2 characteristics.

### CD11b-Enriched Brain-Derived Cells from SIV-Infected Meth-Exposed Animals Exhibit a Mixed M1/M2 Phenotype in Correlation with the Elevation of IL15

**FIGURE 5:**
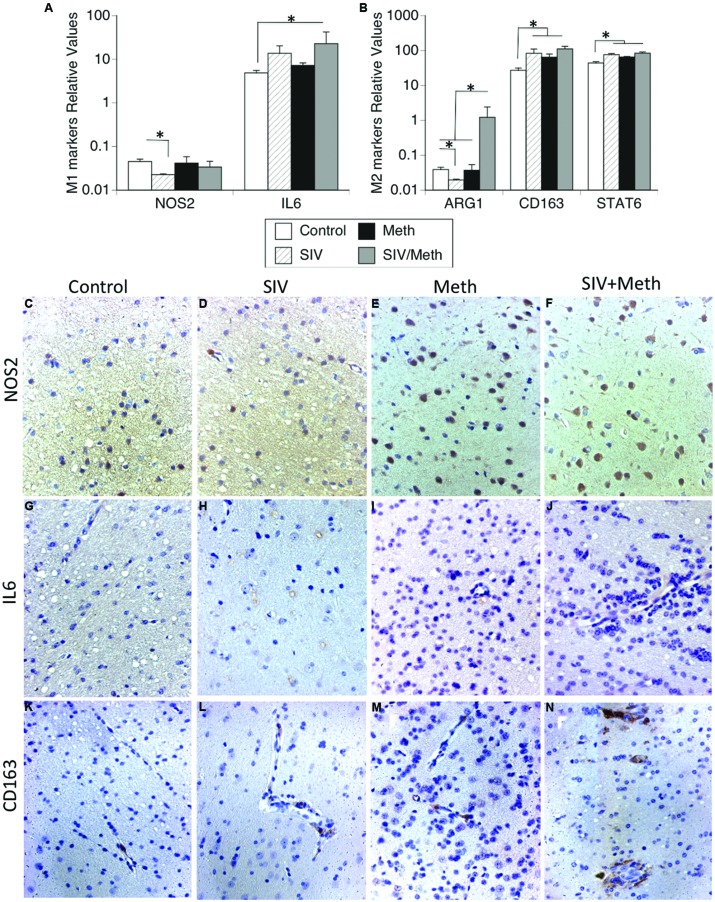
**Detection of functional M1/M2 phenotypic markers in mononuclear cells isolated from the brain and in brain tissue.** Isolated cells and fixed brain tissue from uninfected controls, or SIV-infected, or Meth-treated, or SIV-infected and Meth-treated macaques were used to detect M1 and M2 functional markers. **(A)** Transcriptional levels of M1 functional markers NOS2 and IL6. **(B)** Transcriptional levels of M2 functional markers Arg1, CD163, and Stat6 in isolated brain-derived mononuclear cells from macaques’ brains. Results represent the average ± SEM of three technical replicates. ^∗^*p* ≤ 0.05 in comparisons shown in lines (one-way ANOVA, followed by Bonferroni’s *post hoc* test). Immunohistochemistry on paraffin-embedded pre-frontal cortex from **(C,G,K)** Uninfected controls, **(D,H,L)** SIV-infected, **(E,I,M)** Meth-treated, and **(F,J,N)** SIV-infected Meth-treated macaques, showing the protein expression and distribution of **(C–F)** NOS2, **(G–J)** IL6 and **(K–N)** CD163, from one representative animal in each experimental group, at 26x magnification.

Isolated brain mononuclear cells from SIV-infected Meth-treated macaques had a significantly higher transcriptional expression of IL6 (**Figure 5A**) and Arg1 (**Figure 5B**), compared to control animals. The transcription of the M1 marker NOS2 was not affected by Meth or SIV (**Figure 5A**). The expression of M2 markers CD163 and Stat6 was increased by Meth and by SIV alone, as well as by SIV and Meth together (**Figure 5B**). This suggests that brain-derived mononuclear cells from Meth alone or SIV alone exhibit an M2 functional pattern, whereas the combination of SIV and Meth shows a mixed M1/M2 functional phenotype.

At the protein level, immunohistochemical studies showed that there is increase on NOS2-positive cells both with Meth and with SIV plus Meth (**Figures 5E,F**, respectively), but not with SIV alone (**Figure 5D**), when compared to controls (**Figure 5C**). However, the cells that become NOS2-positive do not have morphological characteristics of microglia or macrophages, but instead share morphological characteristics with neurons. We also examined the expression of IL6 in the brain tissue. We observed that in controls, IL6-positive cells are rare (**Figure 5G**), while in SIV-infected macaques IL6-positive cells are abundant, especially located in layer V of the frontal cortex, in intracellular vesicular structures (**Figure 5H**). Such IL6-stained cell structures can be also found in Meth-treated (**Figure 5I**), and in SIV plus Meth. In the latest, endothelial cells also become lightly positive to IL6 (**Figure 5J**). Regarding the levels and distribution of CD163 (**Figures 5K–N**), we noted that the cells that were CD163-positive were located predominantly in the perivascular domain. In controls, CD163-positive cells were rare, but detectable (**Figure 5K**). There was an discrete increase in the frequency of CD163 in SIV-infected (**Figure 5L**) and in Meth-treated brains (**Figure 5M**) when compared to controls. However, animals that were both SIV-infected and Meth-treated showed a higher number of CD163-positive cells, which were more abundant around vessels and were also found in the brain parenchyma (**Figure 5N**), suggesting that although transcriptional levels of CD163 were similar between SIV/Meth, SIV and Meth-alone, post-transcriptional mechanisms are enhanced by Meth in SIV-infected brains.

Overall, these results suggest that Meth, in the context of SIV infection, alters the immune environment in the CNS by increasing the transcription of IL2RG and of its binding cytokines IL4, IL7, IL15. Among these cytokines, IL15 showed a stronger *in vitro* capacity to increase adhesion molecules, activation markers, and the proliferation rate on macrophages, as well as to modify opposing innate immune functional phenotypes.

## Discussion

Cytokines utilizing the IL2RG have typically been studied regarding their ability to interfere with activation, survival, and homeostasis of T cells (Rubinstein et al., 2008; Sprent et al., 2008; Surh and Sprent, 2008). Especially in the context of neuroAIDS, we have reported the involvement of IL15 in maintaining the presence of activated CD8 T cells chronically in the CNS (Marcondes et al., 2007). Here, we have shown that, in a model of Meth abuse in rhesus macaques, IL2RG is the most significantly upregulated gene in innate immune cells isolated from the brain, regardless of SIV infection. This suggests a role for this molecule in the neuropathology that is developed by Meth use. In addition, in the presence of SIV, cytokines that utilize the IL2RG including IL4, IL7, and IL15 are also upregulated by Meth compared to SIV only. Nevertheless, in Meth-treated SIV-infected macaques the number of CD8 or CD4 T cells in the brain is similar to SIV-only macaques, although a higher inflammatory content, characterized by a higher number of macrophages, microglia activation and astrogliosis, was observed (Marcondes et al., 2007). Thus, we investigated the possibility that these cytokines play a role in the development of inflammation by acting on macrophages and potentially on microglia cells. We found that, in the THP1 macrophage cell line, IL4, IL7, and IL15 have the capacity to increase the expression of molecules such as CD11a and CD11b, which are activation markers and that play a role in adhesion, opsonization, phagocytosis, and chemotaxis (Lo et al., 1989). These cytokines did not affect the expression of HIV-relevant chemokine receptors such as CCR5, or subset markers, suggesting the selective character of the IL2RG usage and the potential effect of other receptors of its ligands. Interestingly, however, IL4, IL7, and IL15 were able to increase the expression of Ki-67 in macrophage cell lines, suggesting that these cytokines have the ability to trigger and to increase the proliferation rate. Meth also enhanced the transcription of functional markers indicating opposing phenotypes in innate immune cells, such as IL6 and CD163.

By immunohistochemistry in the macaques’ brains, the increase in IL6 was clear in all treated groups. On the other hand, the increase of CD163 was only discrete in Meth or SIV-infected animals, but highly enhanced in SIV-infected animals that were also Meth-treated, compared to controls. In perspective with transcriptional data from macaques’ brain-derived mononuclear cells, where there were no differences in CD163 levels between SIV-infected, Meth-treated or SIV/Meth animals, this suggests that Meth may have an action in post-transcriptional mechanisms, further enhancing the phenotypic shift. Additional experiments will be necessary using brain-derived primary cultures to confirm the role of these cytokines in driving proliferation and enhancing selective aspects of the inflammatory phenotype, both transcriptionally and at the protein level.

The IL2RG cytokine system plays a role in determining the character of the local immune environment, in the brain and elsewhere. For instance, it has been reported that activation of macrophages via IL4 can induce their proliferation locally (Jenkins et al., 2011). In addition, macrophages activated by IL4 exhibit an M2 phenotype (Loke et al., 2002), which produce immune regulatory factors, such as IL10 and TGFβ1 that dampen the immune response. In an Alzheimer model, short-term IL4 expression increases amyloid deposition, likely through decreased ability of glial cells to scavenge debris (Chakrabarty et al., 2012). Conversely, IL15 has been suggested to be an autocrine regulator of macrophage functions, which is able to induce TNFα and other pro-inflammatory markers, such as iNOS and IFNb (Alleva et al., 1997; Liu et al., 2004). In addition, IL15 has a role in preserving NK homeostasis and activity (Ranson et al., 2003a,b), which is in agreement with our previous findings of enhanced NK activity in the brain of Meth-treated SIV-infected macaques (Marcondes et al., 2010). Whether the functions of IL4 and IL15 in macrophages depend on signaling through IL2RG or other receptors remains unclear.

Furthermore, these cytokines can contribute to the development of CNS pathology through their actions on neurons. For instance, we have recently reported that IL4 can be an important component leading to selective loss of dopaminergic neurons upon usage of IL13Rα1 expressed on DA cells (Morrison et al., 2012). The increase of IL4 in the brain of Meth-treated animals, regardless of SIV infection, can therefore offer an explanation for a high incidence of Parkinson’s disease among drug users (Thrash et al., 2009).

Cerebral IL15 has also been regarded as a cytokine with implications in neuropathology and behavior. For instance, IL15 is correlated to astrogliosis, and promotes the activation of MAPK and NFkB (Gomez-Nicola et al., 2008a,b, 2010a,b). Conversely, IL15 has anti-apoptotic and neurotrophic effects, due to its ability to suppress nitric oxide in neurons (Budagian et al., 2006), which can be one of the components responsible for its protective effects in experimental autoimmune encephalomyelitis (Gomez-Nicola et al., 2010a). In contrast, IL15-producing astrocytes are a characteristic of demyelinating MS lesions, potentially contributing to CD8 effector functions (Saikali et al., 2010). Thus, IL15 can be potentially beneficial, although it has been associated to neurodegeneration. In LPS models, the pro-inflammatory actions of IL15 may be through IL2RG, which is highly upregulated in several areas of the brain such as hypothalamus and striatum, while the increase on IL15RA seems to be restricted to the hypothalamus (Pan et al., 2013). In EAE, the upregulation of IL2RG and other IL15-binding receptors is widespread (Pan et al., 2013).

IL15 seems to have an important positive impact on mood and memory through IL15RA signaling (Pan et al., 2013). Animals lacking IL15RA show depressive-like behavior and impaired memory, even though they show reduced anxiety (Wu et al., 2011). Interestingly, our data suggests that IL15RA is significantly upregulated by SIV infection. However, the lack of IL2RG does not show the same effects, although a side-by-side comparison was never performed (He et al., 2010; Wu et al., 2010a,b). Thus, IL2RG cytokines can act not only on T cells, but also directly on innate immune cells, as factors that can contribute to the alterations of the CNS environment toward inflammation and dysfunction.

Overall, our data suggests that the ability of Meth to increase IL2RG, and its ligand cytokines in the context of SIV infection, is an important cellular and biochemical mechanism that could contribute to aggravating the neuroinflammatory scenario in HIV-infected patients that are drug users, by driving important phenotypic changes in local innate immune cells.

## Conflict of Interest Statement

The authors declare that the research was conducted in the absence of any commercial or financial relationships that could be construed as a potential conflict of interest.
